# Plants against cancer: towards green Taxol production through pathway discovery and metabolic engineering

**DOI:** 10.1007/s42994-024-00170-8

**Published:** 2024-05-26

**Authors:** Philipp Zerbe

**Affiliations:** Department of Plant Biology, University of California Davis, Davis, CA 95616 USA

**Keywords:** Taxol, Plant natural products, Synthetic biology, Pathway discovery, Metabolic engineering

## Abstract

The diversity of plant natural products presents a rich resource for accelerating drug discovery and addressing pressing human health issues. However, the challenges in accessing and cultivating source species, as well as metabolite structural complexity, and general low abundance present considerable hurdles in developing plant-derived therapeutics. Advances in high-throughput sequencing, genome assembly, gene synthesis, analytical technologies, and synthetic biology approaches, now enable us to efficiently identify and engineer enzymes and metabolic pathways for producing natural and new-to-nature therapeutics and drug candidates. This review highlights challenges and progress in plant natural product discovery and engineering by example of recent breakthroughs in identifying the missing enzymes involved in the biosynthesis of the anti-cancer agent Taxol^®^. These enzyme resources offer new avenues for the bio-manufacture and semi-synthesis of an old blockbuster drug.

## Introduction

Plants are remarkable biochemists; they produce a wealth of bioactive small-molecule chemicals to mediate developmental processes, communicate with other organisms, and adapt to dynamic ecological environments. From the use of yarrow and chamomile by Neanderthalian cultures, nearly 50,000 years ago (Hardy et al. 2012), to traditional medicines developed by ancient civilizations, across the globe, humans have benefitted from the diversity of herbal remedies to treat ailments and diseases. Today, almost a third of World Health Organization essential medicines find their origin in plant natural products (De Luca et al. 2012; Raskin et al. 2002). Faced with increasing health challenges of a burgeoning world population, the large-scale production of clinically used therapeutics and the discovery of new drug leads are a matter of utmost urgency.

## Traditional production approaches

The diversity of plant natural products can provide a critical resource in this endeavor if challenges toward their production can be overcome. Traditional methods of isolating bioactive products from the source species have laid the foundation for many modern pharmaceuticals (Hartmann 2007). However, this strategy is often impractical, due to the lack of scalable cultivation, low product accumulation often in only specific tissues and in response to environmental stimuli, and the need to protect rare and endangered species. Over the past century, chemical synthesis has successfully provided high-purity therapeutics and other bioproducts (Guerra-Bubb et al. 2012; Hetzler et al. 2022), but is often constrained by high costs, the toxicity of waste products, and the structural complexity of plant natural products.

## The transition to modern omics approaches: Taxol as a case study

The availability of large omics data, paired with inexpensive DNA synthesis, has revolutionized the discovery of enzymes and pathways underlying the biosynthesis of desired products (Li et al. 2023; Owen et al. 2017; Tiedge et al. 2020). Applying this resource to metabolic engineering, in heterologous microbial or plant platforms, now offers unprecedented possibilities for manufacturing natural and new-to-nature bioproducts with superior stereo-control and at an industrial scale using enzymatic and semi-synthetic approaches (Chen et al. 2024; Owen et al. 2017; Wurtzel and Kutchan 2016).

The diterpenoid anti-cancer drug Paclitaxel (Taxol^®^) exemplifies the various bottlenecks one can encounter in producing plant-derived therapeutics. Following its discovery in a drug screen of more than 100,000 plant natural products in the late 1960s, Taxol quickly became a leading chemotherapeutic, due to its unique mode of action in arresting mitosis and ultimately cell division by preventing microtubule disassembly and its broad-spectrum activity against several cancer types (Arnst 2020).

**Fig. 1:**
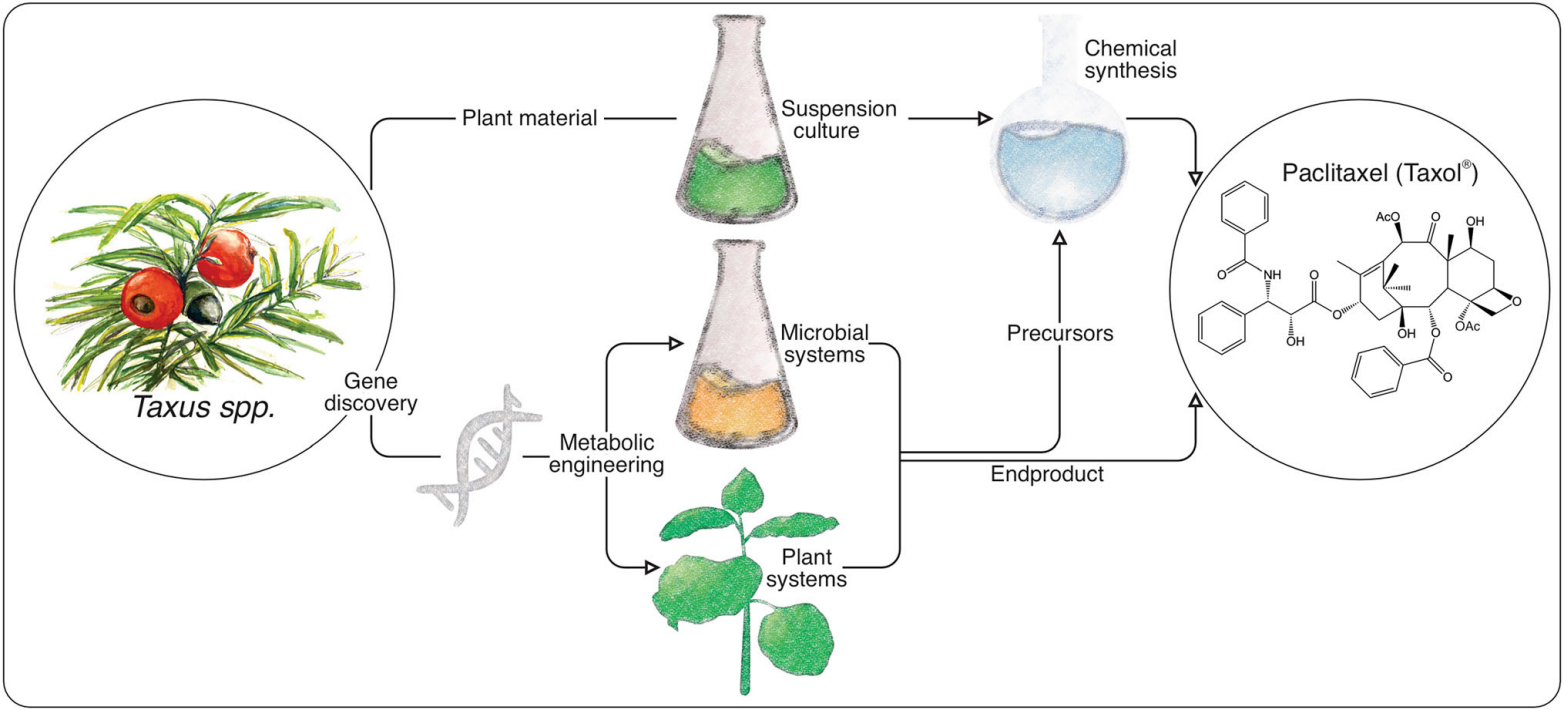
Current strategies for plant-derived Taxol production include the formation of key precursors in *Taxus* cell suspension cultures followed by chemical synthesis of Taxol. Alternatively, the discovery and engineering of Taxol-biosynthetic enzymes in microbial or plant host systems enables Taxol production, or relevant precursors that can be further converted by chemical synthesis

Taxol was first isolated from its natural source, Pacific yew (*Taxus brevifolia*) (Wani et al. 1971). However, coniferous yew trees do not present a sustainable resource, as they grow slowly and in only narrow climatic niches and produce only low amounts of Taxol. Moreover, the isolation of Taxol from bark tissue is destructive, which resulted in overharvesting to the extent that some yew species, historically used for commercial extraction, have been placed on the endangered species list by the International Union for Conservation of Nature (Mayor 2011). This limited natural supply chain has inspired many efforts, over the past six decades, to devise Taxol production strategies that can meet ever-increasing clinical demand (Fig. 1).

Numerous total synthesis routes for Taxol and key precursors have been established, but often require long and expensive routes due to the structural complexity of Taxol (Guerra-Bubb et al. 2012; Watanabe et al. 2023; Zhang et al. 2023a). Early work on the Taxol biosynthetic pathway facilitated the development of cell suspension cultures of *Taxus* needles to produce key precursors, such as 10-deacetyl-baccatin III with high stereochemical precision (Hezari et al. 1997; Ketchum et al. 2007, 2003). Semi-synthetic approaches, utilizing these precursors as starting material, have proven a more renewable and scalable strategy and are currently the major platform for commercial Taxol production (Arya et al. 2020; Roberts 2007).

Rapid advances in synthetic biology have the potential to offer less costly and more sustainable avenues for Taxol manufacture but necessitate knowledge of the underlying enzymes and pathways. Such resources would not only enable improved Taxol production in existing and new metabolic engineering and semi-synthetic platforms but also facilitate combinatorial metabolic engineering of Taxol-biosynthetic enzymes, to thereby gain access to a broader range of the more than 600 known taxane and taxoid structures with potentially desirable therapeutic efficacies (Lange and Conner 2021).

**Fig. 2:**
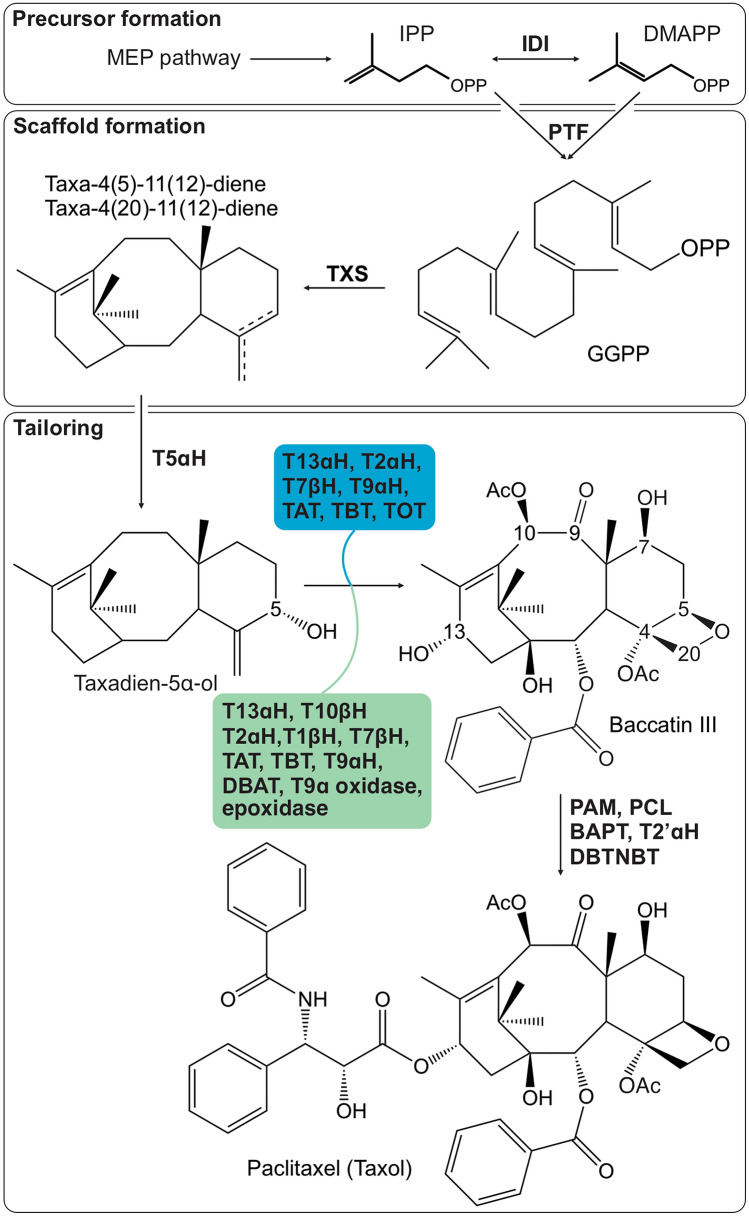
Schematic overview of Taxol biosynthesis. Rooted in the methyl-erythritol phosphate (MEP) pathway, the universal diterpenoid precursor, geranylgeranyl diphosphate (GGPP) is converted into the taxadiene isomers by the diterpene synthase, taxadiene synthase (TXS). Further functional decoration of the taxane scaffold, through members of the cytochrome P450, dioxygenase, transferase and ligase superfamilies, yields the key intermediate baccatin III and, ultimately Taxol. The minimal set of enzymes used for engineering of baccatin III production in Nicotiana benthamiana by Jiang and colleagues (Jiang et al. 2024) and Zhang and coworkers (Zhang et al. 2023b) are highlighted in blue and green boxes, respectively

These applications have steered long-standing research efforts in elucidating the multi-enzyme Taxol-biosynthetic pathway. In particular, the pioneering work by Croteau and colleagues resulted in the discovery of many of the core reactions and associated enzymes of Taxol formation (Guerra-Bubb et al. 2012; Jennewein and Croteau 2001; Walker and Croteau 2001) (Fig. 2). This includes the diterpene synthase, taxadiene synthase (TXS), catalyzing the conversion of the universal diterpenoid precursor, geranylgeranyl diphosphate (GGPP), into taxadiene as the committed reaction in building the core taxane scaffold (Hezari et al. 1995; Lin et al. 1996; Wildung and Croteau 1996). In later years, several cytochrome P450 monooxygenases (P450) and acyl- and benzoyl-transferases, which functionally decorate taxadiene, were characterized (Guerra-Bubb et al. 2012; Srividya et al. 2020).

Equipped with this pathway knowledge, the metabolic engineering of taxol precursors, especially taxadiene and taxadiene-5ɑ-ol, using microbial and plant platforms could be established, including yeast (*Saccharomyces cerevisiae*) (Nowrouzi et al. 2024, 2020; Walls et al. 2021), *Escherichia coli* (Ajikumar et al. 2010; Biggs et al. 2016; Chang et al. 2007) and *Nicotiana benthamiana* (De La Peña and Sattely 2021; Hasan et al. 2014; Li et al. 2019). Notably, product yields varied across studies and host systems, as exemplified by taxadiene titers reaching 103 mg L^−1^ in yeast, 1 g L^−1^ in *E. coli*, and 100 µg g^−1^ fresh weight in *N. benthamiana*. Despite these many advances, several enzymes essential for forming the core baccatin III intermediate, alongside additional modifications and formation of the complete aromatic side chain have remained elusive until recently.

What has been the challenge? Low abundance, diversity, and structural complexity of naturally occurring taxanes and related structures in species of *Taxus* have hindered access to pathway intermediates required for the biochemical testing of enzyme functions. Even if substrates are available, the functional diversity and catalytic promiscuity of the large P450 and transferase enzyme families predicted to be involved in Taxol formation, along with often low protein expression and activity in heterologous systems, has slowed progress in identifying pathway enzymes and understanding the pathway organization of Taxol biosynthesis.

## *Taxus* genomes: platform for missing enzyme discovery

Sequencing and assembly of the > 10 Gb genomes of two *Taxus* species provided a critical milestone in the quest for Taxol biosynthesis (Cheng et al. 2021; Xiong et al. 2021). Recent efforts have leveraged this resource to identify the missing enzymes and reactions to complete the Taxol pathway, marking a significant breakthrough in our understanding and metabolic engineering of Taxol biosynthesis. Integrating genomics, synthetic biology, and enzyme biochemical approaches, Jiang and coworkers elegantly elucidated two P450 enzymes that catalyze two previously unresolved functional modifications essential for Taxol bioactivity (Jiang et al. 2024) (Fig. 2).

Mining of the *Taxus* genomes revealed new members of the *Taxus*-specific CYP725 P450 family with known functions in Taxol formation (Kaspera and Croteau 2006). To enable P450 functional testing, these authors combined co-infiltration of substrate isolated from plant tissue with co-expression of P450 candidates and known pathway enzymes in *N. benthamiana* and insect cell cultures. This strategy resulted in the identification of Taxane Oxetanase 1 (TOT), a bifunctional CYP725 P450 that facilitates the addition of the characteristic oxetane ring to the taxane scaffold (Jiang et al. 2024) (Fig. 2). Functional analysis of insect microsomal fractions containing TOT and knock-down of TOT in *Taxus* cell cultures verified TOT functionality.

Next, Jiang et al. (2024) employed pathway engineering to generate the alternate intermediate, taxusin, and used this platform to functionally screen the remaining CYP725 candidates, leading to the discovery of taxane-9ɑ-hydroxylase (T9ɑH) that catalyzes the missing oxygenation at the C-9 position. With these enzymes in hand, the authors reconstituted the conversion of the universal diterpenoid precursor, geranylgeranyl diphosphate (GGPP), into baccatin III in *N. benthamiana*, using a subset of nine enzymes (Fig. 2), thus, paving the way for the scalable production of key Taxol precursors, through metabolic engineering. Strikingly, some enzymes, such as T10βH and DBAT, which were previously shown to catalyze reactions in Taxol biosynthesis, were not required to form baccatin III.

Other recent studies have demonstrated that species of *Taxus* feature different enzymes and pathway branches to form Taxol and key intermediates. Zhang and colleagues engineered taxadiene production using cytosolic re-localization of the plastidial enzymes in *N. benthamiana*, reaching yields of 100 µg g^−1^ fresh weight that enabled the analysis of additional candidate genes identified in the *Taxus* genome (Zhang et al. 2023b). This approach uncovered T9ɑH (designated as CYP725A22-1 in this study), as well as two 2-oxoglutarate-Fe(II)-dependent dioxygenases that catalyze the oxidation of the C-9 hydroxyl group and formation of the oxetane ring, respectively, thus providing a separate set of 13 enzymes enabling these critical functional decorations of the Taxol backbone (Fig. 2). Further characterization of a T2’ɑH P450 enzyme (CYP73A171) and a β-phenylalanine-CoA ligase, with functions in forming the aromatic side chain, enabled the complete production of Taxol in *N. benthamiana* with a set of 17 enzymes (Zhang et al. 2023b).

**Fig. 3:**
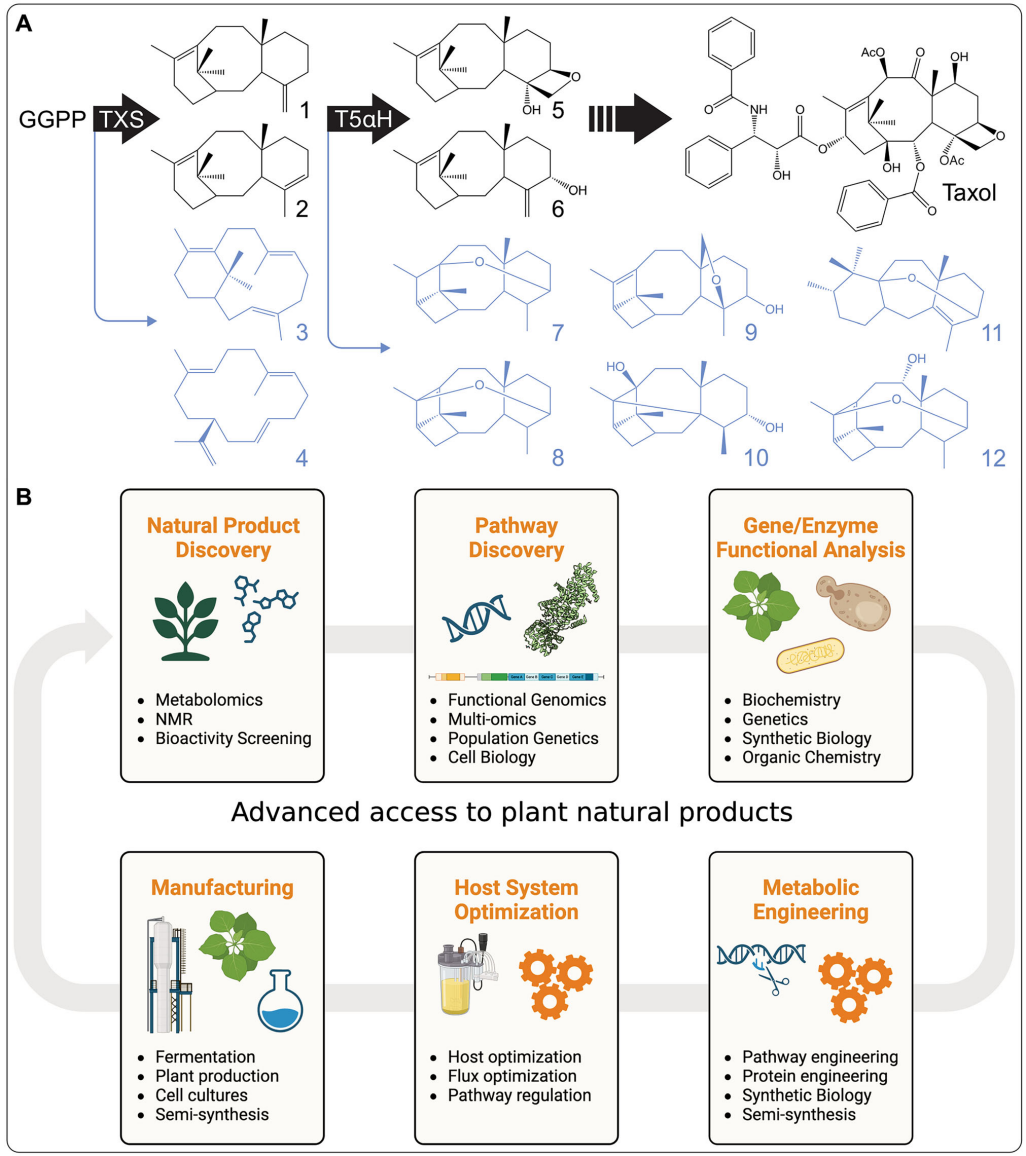
**A** Enzyme catalytic promiscuity in Taxol biosynthesis exemplified by the product diversity of taxadiene synthase (TXS) and taxane 5ɑ-hydroxylase (T5ɑH) toward intermediates *en route* to Taxol (black) and various other taxane structures (blue). **B** Integration of present systems and synthetic biology technologies can drive the discovery, characterization, and engineering of plant natural product pathways, in almost any species. (1) taxa-4(20),11(12)-diene, (2) taxa-4(5),11(12)-diene, (3) verticillia-3(4),7(8),11(12)-triene, (4) cembrene A, (5) 4-hydroxy-5,20-epoxy-taxane, (6) taxadien-5ɑ-ol, (7) iso-OCT, (8) 5(12)-oxa-3(11)-cyclotaxane (OCT), (9–12) additional T5ɑH products (Liu et al. 2024)

Furthermore, Yang et al. also reported the characterization of T9αH (here designated CYP725A37) as well as the CYP725A55-catalyzed oxetane ester formation to form 1*β*-dehydroxy-baccatin VI (Yang et al. 2024). Motivated by a close review of previously proposed pathway reactions, another recent effort by Zhao and colleagues demonstrated that taxane 5ɑ-hydroxylase (T5ɑH, CYP725A4), a P450 identified to decorate the taxadiene scaffold at C-5 nearly three decades ago (Hefner et al. 1996), can act as a bifunctional enzyme, facilitating C-5 hydroxylation of two primary TXS products, taxa-4(5)-11(12)-diene and its isomer taxa-4(20)-11(12)-diene, and subsequent oxetane ring formation, as shown by engineering taxadiene production and T5ɑH co-expression in yeast and *N. benthamiana* (Zhao et al. 2024) (Fig. 3). In addition, Liu and coworkers combined promoter engineering with co-expression analysis to identify several previously unresolved products of T5ɑH, underscoring the functional promiscuity of this core P450 in the production of Taxol and other taxoids (Liu et al. 2024) (Fig. 3).

## Catalytic plasticity: dynamic metabolic networks

These research advances not only discovered long sought-after pathway reactions in Taxol biosynthesis but, more broadly, highlight the potential of integrating systems biology, synthetic biology, and modern metabolomics and biochemical technologies to realize the discovery and engineering of multi-enzyme pathway networks *en route* to highly complex specialized metabolites that previously were unattainable. Notably, several findings suggest that similar to other diterpenoid pathways, the biosynthesis of Taxol and related taxoids is realized through a dynamic metabolic network, where individual enzyme modules can interact in different combinations to yield a broader product range (Bathe and Tissier 2019; Lanier et al. 2023; Peters 2006; Zerbe and Bohlmann 2015).

Firstly, the above-mentioned studies revealed that different enzymes are capable of generating the signature oxetane ring critical for the therapeutic efficacy of Taxol (Wang et al. 2000). Secondly, differences in the tissue-specific expression of several identified genes support the presence of tissue-specific pathways (Jiang et al. 2024). Thirdly, TXS, T5ɑH, and other Taxol-forming enzymes show expansive substrate- and product-promiscuity (Guerra-Bubb et al. 2012; Liu et al. 2024; Zhao et al. 2024) (Fig. 3), thus supplying substrates for alternate pathway branches toward the diverse array of taxanes and taxoids produced in species of yew (Lange and Conner 2021). Notably, the use of different minimal enzyme sets to produce baccatin III in *N. benthamiana* (Fig. 2) resulted in different product yields between 50 ng g^−1^ (Jiang et al. 2024) and 155 ng g^−1^ (Zhang et al. 2023b) plant material, suggesting that differences in enzyme combinations and pathway reconstitution affect pathway productivity.

The catalytic plasticity of Taxol biosynthesis presents both a challenge and an opportunity for metabolic engineering. Combinatorial pathway engineering of different enzyme modules can provide access to a range of structures, whereas lack of control over undesired branch pathways can substantially diminish product yield in heterologous systems (Andersen-Ranberg et al. 2016; De La Peña and Sattely 2021; Frey et al. 2024; Guo et al. 2016; Liu et al. 2024; Mafu et al. 2016). Although production yields of baccatin III and Taxol in yeast and *N. benthamiana* are still relatively low, advances in multi-enzyme pathway engineering, subcellular co-localization of enzyme modules, and engineering of microbial and plant host systems now offer the tools needed for developing large-scale Taxol production platforms (Jiang et al. 2024; Zhang et al. 2023b).

By integrating genomics-enabled gene discovery, enzyme co-expression approaches, and substrate feeding, a broader range of precursors can be accessed to fast-track the functional testing and annotation of enzyme superfamilies involved in the biosynthesis of all classes of plant-specialized metabolites (De La Peña and Sattely 2021; Frey et al. 2024; Kitaoka et al. 2015; Tiedge et al. 2020) (Fig. 3). To optimize pathway engineering toward Taxol and other desired products, a fundamental knowledge of the order of enzyme reactions and the spatial/temporal organization of pathways is required to enable the redirection of precursor flux and control enzyme expression levels, in heterologous systems that lack the native regulatory components (Ajikumar et al. 2010; Liu et al. 2024; Nowrouzi et al. 2024; Zhao et al. 2024). Complementary to pathway discovery and optimization discussed here, advances in metabolic engineering, fermentation, and plant biomass production, as well as semi-synthetic approaches are certain to continue boosting natural product titers in microbial and plant platforms (Wang et al. 2021; Biggs et al., 2021; Belcher et al. 2021). At the same time, rapid advances in metabolomics technologies enable the screening of a broad range of species across the plant kingdom and are certain to reveal new bioactive natural products as leads for drug discovery.

## Conclusions

Continued efforts to decipher the structure–activity relationships of Taxol-biosynthetic enzymes will enable protein engineering to improve catalytic activity and specificity (Biggs et al. 2016; Edgar et al. 2016; Köksal et al. 2011; Liu et al. 2024; Schrepfer et al. 2016; You et al. 2018). Ultimately, combining the expansive tool kit, at the interface of modern biology and chemistry, can accelerate the discovery and sustainable manufacture of life-saving chemicals powered by plants.

## Data Availability

Data sharing is not applicable to this article as no datasets were generated or analyzed as part of this study.
